# Association and prediction of red blood cell distribution width to albumin ratio in all-cause mortality of acute kidney injury in critically ill patients

**DOI:** 10.3389/fmed.2023.1047933

**Published:** 2023-03-09

**Authors:** Chen Gao, Longkai Peng

**Affiliations:** Department of Kidney Transplantation, The Second Xiangya Hospital of Central South University, Changsha, China

**Keywords:** RDW/ALB, all-cause mortality, AKI, critically ill, MIMIC-III

## Abstract

**Aim:**

The progression of acute kidney injury (AKI) might be associated with systemic inflammation. Our study aims to explore the association and predictive value of the red blood cell distribution width (RDW) to human serum albumin (ALB) ratio (RDW/ALB ratio), an inflammation-related indicator, in the risk of all-cause mortality and renal replacement therapy (RRT) in AKI patients admitted in intensive care units (ICU).

**Methods:**

A retrospective cohort study was designed, and data were extracted from the Medical Information Mart for Intensive Care III (MIMIC-III). The primary outcome was the risk of all-cause mortality (1-month, 3-month, and 12-month), and the secondary outcome was the risk of RRT. The association between the RDW/ALB ratio and the risk of all-cause mortality and RRT was assessed using the Cox regression analysis, with results shown as hazard ratio (HR) and 95% confidence intervals (CIs). The relationship between the RDW/ALB ratio and crude probability of all-cause mortality or RRT was assessed using restricted cubic splines (RCS). The concordance index (C-index) was used to assess the discrimination of the prediction model.

**Results:**

A total of 13,856 patients were included in our study. In the fully adjusted Cox regression model, we found that a high RDW/ALB ratio was associated with an increased risk of 1-month, 3-month, and 12-month all-cause mortality and RRT (all *p* < 0.05). Moreover, RCS curves showed the linear relationship between the RDW/ALB ratio and the probability of all-cause mortality and RRT, and the probability was elevated with the increase of the ratio. In addition, the RDW/ALB ratio showed a good predictive performance in the risk of 1-month all-cause mortality, 3-month all-cause mortality, 12-month all-cause mortality, and RRT, with a C-index of 0.728 (95%CI: 0.719–0.737), 0.728 (95%CI: 0.721–0.735), 0.719 (95%CI: 0.713–0.725), and 0.883 (95%CI: 0.876–0.890), respectively.

**Conclusion:**

The RDW/ALB ratio performed well to predict the risk of all-cause mortality and RRT in critically ill patients with AKI, indicating that this combined inflammatory indicator might be effective in clinical practice.

## Introduction

Acute kidney injury (AKI) is a syndrome characterized by a sudden deterioration of kidney function (1). AKI occurs in 10–15% of all patients admitted to the hospital (2), and its prevalence exceeds 50% in intensive care unit (ICU) patients (3). Three to five-fold greater mortality was reported in critically ill patients with AKI compared to those without AKI (4). In the United States, 6–24% of ICU patients experience AKI, whose short-term all-cause mortality is as high as 60–70% (5, 6), which substantially elevates healthcare costs and brings great healthcare burdens. Therefore, it is important to search for valuable indicators to predict the prognosis of critically ill patients with AKI.

The progression of AKI might be associated with systemic inflammation (7). Understanding indicators underlying the inflammatory response has great potential for identifying effective therapies to prevent or improve AKI (7). Red cell distribution width (RDW) is an indicator reflecting the size change of red blood cells, and an increase in RDW is regarded as anisocytosis (8). Studies have reported a strong relationship between inflammatory reactions and an increase in RDW (9, 10). RDW has been reported to be associated with the progressions and outcomes of many diseases, including kidney diseases (9). Wang et al. (11) found that RDW was independently correlated with the short-term mortality of critically ill patients with AKI. Jia et al. (12) reported a positive correlation between RDW to long-term mortality in AKI patients. Also, Zhu et al. (13) found that a high level of RDW could independently predict the need for renal replacement therapy (RRT) in AKI patients. A study has shown that RDW combined with other viable indicators is more accurate in predicting the prognosis of critically ill patients with AKI than RDW alone (14).

Albumin (ALB), synthesized in the liver, is an indicator that reflects nutritional status and participates in regulating inflammatory response (15, 16). Previous studies have reported the association between ALB and AKI, and ALB level bears an inverse association with all-cause mortality in AKI patients (17, 18). Critical illness is usually correlated with systemic inflammation, and the RDW/ALB ratio has been reported as a valuable indicator to predict the prognosis of ICU patients suffering from stroke, acute respiratory distress syndrome, and pneumonia (19–21). However, the clinical utility of the RDW/ALB ratio has not yet been evaluated in critically ill patients with AKI.

This study aimed to explore the association between the RDW/ALB ratio and the prognosis of AKI patients admitted in the ICU, and further explore the predictive value of the RDW/ALB ratio for the prognosis of AKI.

## Methods

### Study design and data source

This retrospective cohort study was performed in 2013, based on the revised Declaration of Helsinki. Data were extracted from the Medical Information Mart for Intensive Care III (MIMIC-III), a public and freely available databasethat included health-related data on over 40,000 patients admitted to the ICU of the Beth Israel Deaconess Medical Center (Boston, MA, United States) from 2001 to 2012 (22). This database preserved extensive clinical data, including demographic characteristics, vital signs, laboratory measurements, medication, and medical records. The MIMIC-III database has obtained approval from the Institutional Review Boards of Beth Israel Deaconess Medical Center (Boston, MA, United States) and the Massachusetts Institute of Technology (Cambridge, MA, United States). To protect patients’ privacy, the personal information of all included patients was de-identified; therefore, this study was exempt from the informed consent of patients.

### Participants

Participants who were aged ≥18 years, met the diagnosis of AKI, and were hospitalized in the ICU within 48 h of first admission (considering one of the AKI definitions was evaluated within 48 h) were included in this study. Participants missing data on ALB and RDW were excluded from this study. The participants were followed up until death after ICU admission.

AKI was defined as an increase of serum creatinine (SCr) level by **≥** 0.3 mg/dl (≥ 26.5 μmol/l) within 48 h or an increase of SCr to ≥ 1.5 times the baseline, which was known or presumed to have occurred within the prior 7 days, or urine volume < 0.5 ml/kg/h for 6 h based on the Kidney Disease: Improving Global Outcomes (KDIGO) (23).

### Data extraction

Data were extracted based on five aspects: baseline characteristics, vital signs, laboratory parameters, scoring systems, and outcome.

Baseline characteristics included age, gender, ethnicity, height, weight, AKI stage, therapy [mechanical ventilation, vasopressors, and renal replacement therapy (RRT)], comorbidities [atrial fibrillation (AF), congestive heart failure (CHF), respiratory failure, diabetes, and hypertension], estimated glomerular filtration rate (eGFR), causes of AKI (sepsis, acute pancreatitis, cardiogenic shock, acute cerebrovascular disease, and liver cirrhosis), ICU length of stay, and hospital length of stay. The eGFR (mL/min/1.73 m) was estimated as 141 × min(Scr/κ, 1)^α^ × max(Scr/κ, 1)^-1.029^ × 0.993^age^ × 1.018 [if female] _ 1.159 [if Black], where Scr was expressed in mg/dL, κ was 0.7 for females and 0.9 for males, *α* was − 0.329 for females and − 0.411 for males, min indicated the minimum of Scr/κ or 1, and max indicated the maximum of Scr/κ or 1 (24).

Vital signs included heart rate, systolic blood pressure (SBP), diastolic blood pressure (DBP), respiratory rate, temperature, saturation of peripheral oxygen (SPO_2_), partial pressure of carbon dioxide (PCO_2_), and partial pressure of oxygen (PO_2_).

Laboratory parameters included white blood cell count (WBC), lymphocytes count, neutrophil count, platelet count (PLT), hemoglobin, hematocrit, creatinine, international normalized ratio (INR), ALB, blood urea nitrogen (BUN), glucose, lactate, bicarbonate, anion gap, RDW, alanine aminotransferase (ALT), aspartate aminotransferase (AST), total calcium, neutrophil-to-lymphocyte ratio (NLR), bilirubin, and prothrombin time (PT).

Scoring systems included Elixhauser Comorbidity Index (ECI), Sequential Organ Failure Assessment (SOFA) score, and Simplified Acute Physiology Score II (SAPSII).

The primary outcome was all-cause mortality (1-month all-cause mortality, 3-month all-cause mortality, and 12-month all-cause mortality), and the secondary outcome was the risk of RRT.

### Cutoff values of RDW/ALB

The optimal cutoff values of the RDW/ALB ratio were obtained using X-tile software (version 3.6.1, Yale University, New Haven, CT, United States) in terms of all-cause mortality and the minimum *p* value method (25, 26). X-tile is a bioinformatics tool to assess biomarkers and optimize outcome-based cut-point values (27). Using X-tile software, two cutoff values (4.6 and 5.9) for the RDW/ALB ratio were obtained (Supplementary Figure S1); therefore, patients were divided into a low ratio group (RDW/ALB < 4.6), a moderate ratio group (RDW/ALB from 4.6 to 5.9), and a high ratio group (RDW/ALB > 5.9).

### Statistical analysis

The normally distributed measurement data were expressed as mean ± standard deviation (mean ± SD), and differences between the two groups were compared using a t test. The non-normally distributed measurement data were presented as median with interquartile range (IQR), and intergroup differences were compared using the Wilcoxon rank sum test. The counting data were shown as the number and percentage [*n* (%)], and the Chi-square test was used to compare differences between groups. Variables (height, weight, PO_2_, bilirubin, and PT) with missing values over 20% were deleted and variables with missing values under 20% were processed using multiple imputations. Sensitivity analysis was conducted before and after imputation. The cutoff values of ALB and RDW were obtained using X-tile software (version 3.6.1). According to the cutoff values, ALB was divided into low level (ALB < 2.6), moderate level (ALB from 2.6 to 3.3), and high level (ALB >3.3); RDW was divided into low level (RDW < 14.4), moderate level (RDW from 14.4 to 15.7), and high level (RDW > 15.7).

Cox regression analysis was used to assess the association between RDW/ALB and the risk of 1-month all-cause mortality, 3-month all-cause mortality, 12-month all-cause mortality, and RRT through stepwise regression (*p* < 0.05 for selection). Results of the cox regression analysis were expressed as hazard ratio (HR) with 95% confidence intervals (CIs). We used different models, including an unadjusted model that was not adjusted for any factors and Model 1 that was adjusted for age, ethnicity, and gender. For all-cause mortality, Model 2 was adjusted for age, ethnicity, AKI stage, heart rate, temperature, SPO_2_, PCO_2_, hematocrit percent, lymphocytes, hemoglobin, creatinine, INR, BUN, lactate, calcium, NLR, bicarbonate, anion gap, AF, CHF, respiratory failure, diabetes, hypertension, ECI, mechanical ventilation, vasopressors, SOFA, SAPSII, RRT, sepsis, acute pancreatitis, liver cirrhosis, and acute cerebrovascular disease. For the risk of RRT, Model 2 was adjusted for age, AKI stage, PCO_2_, lymphocytes, platelet, hematocrit, creatinine, BUN, bicarbonate, calcium, ALT, AST, anion gap, eGFR, NLR, AF, CHF, ECI, SOFA, mechanical ventilation, vasopressors, sepsis, acute pancreatitis, liver cirrhosis, acute cerebrovascular disease, and cardiogenic shock.

Spearman rank correlation coefficient was used to analyze the correlation between RDW/ALB and the indexes of the severity of patients’ conditions and kidney dysfunction (eGFR, SOFA, SAPSII, and AKI stage). Although SOFA and SAPSII were not specific indicators for AKI, they were indicators of the severity of clinical conditions and organ dysfunction at ICU admission (28, 29). AKI stage was the parameter that represented the severity of AKI, and eGFR was an index of baseline kidney function. The discrimination of the prediction model was assessed with the concordance index (C-index). The DeLong test was used to compare differences in the C-index of RDW/ALB, RDW, ALB, SAPSII, and SOFA. Restricted cubic spline (RCS) was only applied to assess the relationship between the RDW/ALB ratio and the crude probability of all-cause mortality and RRT. Kaplan–Meier (KM) method was used to assess the differences in the all-cause mortality of low, moderate, and high RDW/ALB ratio groups based on the log-rank test. Subgroup analysis was performed according to the AKI stage and sepsis (with or without) using multivariate Cox analysis. All data were analyzed by SAS 9.4 (SAS Institute Inc., Cary, NC, United States). KM curves were generated using R version 4.0.3 (R Foundation for Statistical Computing, Vienna, Austria). *p* < 0.05 was considered to be statistically significant.

## Results

### Selection and characteristics of included patients

**Figure 1:**
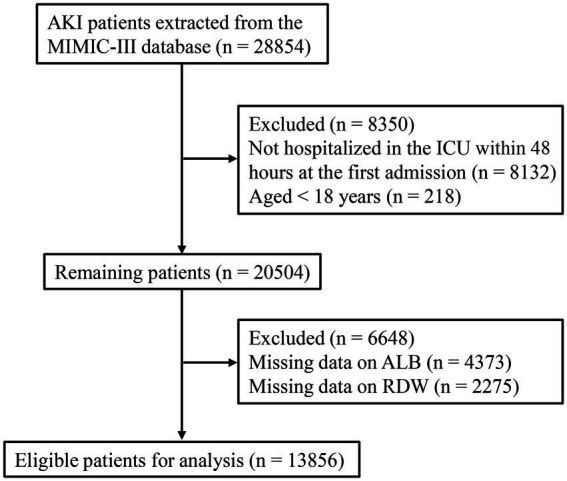
The flowchart of patients’ selection.

A total of 28,854 AKI patients were extracted from the MIMIC-III database. Of these, we excluded 8,132 patients who were not hospitalized in the ICU within 48 h of first admission and 218 patients younger than 18 years old. Of the remaining 20,504 patients, we further excluded those who were missing data on ALB (*n* = 4,373) and RDW (*n* = 2,275). Finally, 13,856 patients were included for analysis (Figure 1). There were 5,816 patients in the low RDW/ALB group, 4,182 patients in the moderate RDW/ALB group, and 3,858 patients in the high RDW/ALB group. The missing data were addressed by multiple imputations, and sensitivity analysis showed consistent results before and after imputation (all *p* > 0.05; Supplementary Table S1). Table 1 displays that age, gender, AKI stage, mechanical ventilation, vasopressors, AF, CHF, respiratory failure, hypertension, eGFR, sepsis, acute pancreatitis, cardiogenic shock, acute cerebrovascular disease, liver cirrhosis, heart rate, SBP, DBP, respiratory rate, temperature, SPO_2_, PCO_2_, WBC, lymphocytes, neutrophil, PLT, hemoglobin, hematocrit, creatinine, INR, ALB, BUN, glucose, lactate, bicarbonate, anion gap, RDW, AST, calcium, NLR, ECI, RRT, SOFA, SAPSII, ICU length of stay, hospital length of stay, RRT, 1-month all-cause mortality, 3-month all-cause mortality, and 12-month all-cause mortality were statistically significant in the low, moderate, and high RDW/ALB groups (all *p* < 0.05).

**Table 1:** Characteristics of the study patients divided by RDW/ALB ratios.

Variables	Total (*n* = 13,856)	RDW/ALB ratio			Statistics	*p*
		Low (*n* = 5,816)	Moderate (*n* = 4,182)	High (*n* = 3,858)		
Age, years	66.17 ± 15.67	66.26 ± 16.07	66.54 ± 15.74	65.66 ± 14.96	*t* = 3.330	0.036
Gender, *n* (%)					*χ*^2^ = 26.179	< 0.001
Female	6,022 (43.46)	2,409 (41.42)	1812 (43.33)	1801 (46.68)		
Male	7,834 (56.54)	3,407 (58.58)	2,370 (56.67)	2057 (53.32)		
Ethnicity, *n* (%)					*χ*^2^ = 5.206	0.267
Black	1,058 (7.64)	439 (7.55)	325 (7.77)	294 (7.62)		
Other/unknown	2,822 (20.37)	1,168 (20.08)	822 (19.66)	832 (21.57)		
White	9,976 (72.00)	4,209 (72.37)	3,035 (72.57)	2,732 (70.81)		
AKI stage, *n* (%)					*χ*^2^ = 436.137	<0.001
1	3,137 (22.64)	1,554 (26.72)	910 (21.76)	673 (17.44)		
2	6,847 (49.42)	3,108 (53.44)	2051 (49.04)	1,688 (43.75)		
3	3,872 (27.94)	1,154 (19.84)	1,221 (29.20)	1,497 (38.80)		
Mechanical ventilation, *n* (%)					*χ*^2^ = 15.193	<0.001
No	2,527 (18.24)	1,117 (19.21)	785 (18.77)	625 (16.20)		
Yes	11,329 (81.76)	4,699 (80.79)	3,397 (81.23)	3,233 (83.80)		
Vasopressors, *n* (%)					*χ*^2^ = 353.587	<0.001
No	7,430 (53.62)	3,557 (61.16)	2,264 (54.14)	1,609 (41.71)		
Yes	6,426 (46.38)	2,259 (38.84)	1918 (45.86)	2,249 (58.29)		
AF, *n* (%)					*χ*^2^ = 20.486	<0.001
No	8,387 (60.53)	3,483 (59.89)	2,456 (58.73)	2,448 (63.45)		
Yes	5,469 (39.47)	2,333 (40.11)	1726 (41.27)	1,410 (36.55)		
CHF, *n* (%)					*χ*^2^ = 9.717	0.008
No	8,171 (58.97)	3,506 (60.28)	2,391 (57.17)	2,274 (58.94)		
Yes	5,685 (41.03)	2,310 (39.72)	1791 (42.83)	1,584 (41.06)		
Respiratory failure, *n* (%)					*χ*^2^ = 188.931	<0.001
No	7,119 (51.38)	3,385 (58.20)	1975 (47.23)	1759 (45.59)		
Yes	6,737 (48.62)	2,431 (41.80)	2,207 (52.77)	2099 (54.41)		
Diabetes, *n* (%)					*χ*^2^ = 4.387	0.112
No	10,018 (72.30)	4,237 (72.85)	2,973 (71.09)	2,808 (72.78)		
Yes	3,838 (27.70)	1,579 (27.15)	1,209 (28.91)	1,050 (27.22)		
Hypertension, *n* (%)					*χ*^2^ = 236.666	<0.001
No	6,569 (47.41)	2,430 (41.78)	1919 (45.89)	2,220 (57.54)		
Yes	7,287 (52.59)	3,386 (58.22)	2,263 (54.11)	1,638 (42.46)		
eGFR, mL/min/1.73 m	64.64 (40.97, 90.04)	70.69 (48.01, 91.50)	60.18 (35.99, 87.02)	61.24 (35.62, 90.38)	*Z* = 169.357	<0.001
Sepsis, *n* (%)					*χ*^2^ = 23.431	<0.001
No	11,045 (79.71)	4,749 (43.00)	3,270 (29.61)	3,026 (27.40)		
Yes	2,811 (20.29)	1,067 (37.96)	912 (32.44)	832 (29.60)		
Acute pancreatitis, *n* (%)					*χ*^2^ = 15.895	<0.001
No	13,216 (95.38)	5,594 (42.33)	3,954 (29.92)	3,668 (27.75)		
Yes	640 (4.62)	222 (34.69)	228 (35.63)	190 (29.69)		
Cardiogenic shock, *n* (%)					*χ*^2^ = 6.980	0.030
No	12,896 (93.07)	5,395 (41.83)	3,875 (30.05)	3,626 (28.12)		
Yes	960 (6.93)	421 (43.85)	307 (31.98)	232 (24.17)		
Acute cerebrovascular disease, *n* (%)					*χ*^2^ = 200.679	<0.001
No	13,212 (95.35)	5,382 (40.74)	4,029 (30.50)	3,801 (28.77)		
Yes	644 (4.65)	434 (67.39)	153 (23.76)	57 (8.85)		
Liver cirrhosis, *n* (%)					*χ*^2^ = 320.104	<0.001
No	12,709 (91.72)	5,576 (43.87)	3,829 (30.13)	3,304 (26.00)		
Yes	1,147 (8.28)	240 (20.92)	353 (30.78)	554 (48.30)		
Heart rate, beats/min	92.75 ± 21.01	90.55 ± 20.19	93.31 ± 20.94	95.45 ± 21.94	*t* = 65.604	<0.001
SBP, mmHg	123.35 ± 27.08	127.34 ± 27.69	122.45 ± 26.50	118.32 ± 25.84	*t* = 134.367	<0.001
DBP, mmHg	62.00 (52.00, 74.00)	64.00 (54.00, 77.00)	62.00 (52.00, 72.00)	59.00 (50.00, 72.00)	*Z* = 178.346	<0.001
Respiratory rate, beats/min	19.00 (14.00, 24.00)	18.00 (14.00, 23.00)	19.00 (14.00, 24.00)	19.00 (15.00, 24.00)	*Z* = 60.588	<0.001
Temperature, °C	36.61 ± 1.11	36.62 ± 1.03	36.65 ± 1.13	36.55 ± 1.20	*t* = 7.237	<0.001
SPO_2_, %	96.80 ± 5.50	97.16 ± 5.18	96.61 ± 5.21	96.46 ± 6.21	*t* = 22.378	<0.001
PCO_2_, mmHg	41.00 (35.00, 49.00)	41.00 (36.00, 48.00)	42.00 (35.00, 49.00)	41.00 (34.00, 49.00)	*Z* = 20.382	<0.001
WBC, 10^3^/μL	10.80 (7.60, 15.70)	10.40 (7.60, 14.10)	11.40 (7.90, 16.60)	11.50 (7.50, 16.80)	*Z* = 91.112	<0.001
Lymphocytes,	9.80 (5.60, 16.00)	11.20 (6.70, 18.90)	9.40 (5.30, 15.00)	8.00 (4.60, 13.30)	*Z* = 484.770	<0.001
Neutrophil,	78.24 ± 14.07	77.76 ± 13.52	78.92 ± 13.75	78.23 ± 15.15	*t* = 8.309	<0.001
PLT, 10^3^/μL	226.00 (161.00, 300.00)	230.00 (179.00, 292.00)	225.00 (158.00, 304.00)	219.00 (126.00, 316.00)	*χ*^2^ = 63.059	<0.001
Hemoglobin, g/dl	11.67 ± 2.25	12.63 ± 2.01	11.28 ± 2.17	10.65 ± 2.10	*t* = 1154.789	<0.001
Hematocrit, %	34.90 ± 6.48	37.40 ± 5.77	33.92 ± 6.38	32.19 ± 6.22	*t* = 927.812	<0.001
Creatinine, mg/dl	1.10 (0.80, 1.70)	1.10 (0.80, 1.50)	1.20 (0.80, 2.00)	1.20 (0.80, 2.00)	*Z* = 144.225	<0.001
INR	1.30 (1.10, 1.60)	1.20 (1.10, 1.40)	1.30 (1.10, 1.60)	1.40 (1.20, 1.80)	*Z* = 727.052	<0.001
ALB, g/dl	3.07 ± 0.70	3.66 ± 0.43	2.94 ± 0.37	2.32 ± 0.45	*t* = 12215.27	<0.001
BUN, mg/dl	23.00 (16.00, 38.00)	21.00 (15.00, 31.00)	25.00 (16.00, 43.00)	27.00 (17.00, 45.00)	*Z* = 376.068	<0.001
Glucose, mg/dl	135.00 (108.00, 177.00)	137.00 (112.00, 183.00)	135.00 (110.00, 175.00)	129.00 (101.00, 171.00)	*Z* = 121.670	<0.001
Lactate, mmol/L,	1.80 (1.30, 2.90)	1.70 (1.20, 2.70)	1.80 (1.20, 2.80)	1.90 (1.30, 3.20)	*Z* = 75.320	<0.001
Bicarbonate, mEq/L,	24.18 ± 5.46	24.85 ± 5.13	24.20 ± 5.48	23.14 ± 5.76	*t* = 115.203	<0.001
Anion gap, mmol/L	15.90 ± 4.49	16.07 ± 4.43	15.99 ± 4.46	15.56 ± 4.60	*t* = 16.399	<0.001
RDW, %	15.14 ± 2.16	14.04 ± 1.18	15.20 ± 1.80	16.76 ± 2.58	*t* = 2533.834	<0.001
ALT, U/L	27.00 (16.00, 54.00)	27.00 (17.00, 49.50)	28.00 (16.00, 58.00)	27.00 (15.00, 57.00)	*Z* = 5.145	0.076
AST, U/L	37.00 (22.00, 77.00)	33.00 (21.00, 64.00)	40.00 (23.00, 83.00)	41.00 (23.00, 89.00)	*Z* = 124.498	<0.001
Calcium, mg/dl	8.44 ± 1.02	8.73 ± 0.90	8.41 ± 0.97	8.03 ± 1.09	*t* = 591.488	<0.001
NLR	8.39 (4.62, 15.38)	7.07 (3.82, 12.94)	8.64 (5.07, 16.11)	10.30 (5.58, 18.71)	*Z* = 389.888	<0.001
ECI	12.00 (5.00, 20.00)	9.00 (3.00, 17.00)	13.00 (7.00, 20.00)	15.00 (8.00, 22.00)	*Z* = 504.724	<0.001
SOFA	7.00 (4.00, 9.00)	6.00 (4.00, 8.00)	7.00 (5.00, 9.00)	8.00 (6.00, 11.00)	*Z* = 573.124	<0.001
SAPSII	42.00 (34.00, 52.00)	39.00 (31.00, 48.00)	43.00 (34.00, 53.00)	46.00 (37.00, 56.00)	*Z* = 598.343	<0.001
ICU length of stay	8.76 (4.01, 21.32)	6.67 (3.43, 17.05)	9.47 (4.27, 21.91)	11.40 (5.08, 25.14)	*Z* = 334.688	<0.001
Hospital length of stay	17.17 (9.74, 29.23)	14.58 (8.67, 25.22)	18.53 (10.45, 29.08)	20.57 (11.20, 33.40)	*Z* = 302.194	<0.001
RRT, *n* (%)					*χ*^2^ = 209.848	<0.001
No	11,514 (83.10)	5,133 (44.58)	3,394 (29.48)	2,987 (25.94)		
Yes	2,342 (16.90)	683 (29.16)	788 (33.65)	871 (37.19)		
1-month all-cause mortality, *n* (%)					*χ*^2^ = 337.715	<0.001
No	11,220 (80.98)	5,072 (87.21)	3,360 (80.34)	2,788 (72.27)		
Yes	2,636 (19.02)	744 (12.79)	822 (19.66)	1,070 (27.73)		
3-month all-cause mortality, *n* (%)					*χ*^2^ = 604.116	<0.001
No	9,588 (69.20)	4,621 (79.45)	2,800 (66.95)	2,167 (56.17)		
Yes	4,268 (30.80)	1,195 (20.55)	1,382 (33.05)	1,691 (43.83)		
12-month all-cause mortality, *n* (%)					*χ*^2^ = 733.826	<0.001
No	7,990 (57.66)	4,071 (70.00)	2,270 (54.28)	1,649 (42.74)		
Yes	5,866 (42.34)	1745 (30.00)	1912 (45.72)	2,209 (57.26)		

AKI, acute kidney injury; AF, atrial fibrillation; CHF, congestive heart failure; eGFR, estimated glomerular filtration rate; SBP, systolic blood pressure; DBP, diastolic blood pressure; SPO_2_, saturation of peripheral oxygen; PCO_2_, partial pressure of carbon dioxide; WBC, white blood cell count; PLT, platelet count; INR, international normalized ratio; ALB, albumin; BUN, blood urea nitrogen; RDW, red blood cell distribution width; ALT, alanine aminotransferase; AST, aspartate aminotransferase; NLR, neutrophil-to-lymphocyte ratio; ECI, Elixhauser Comorbidity Index; SOFA, Sequential Organ Failure Assessment score; SAPSII, Simplified Acute Physiology Score II; ICU, intensive care unit; and RRT, renal replacement therapy.

Low ratio: RDW/ALB < 4.6; moderate ratio: 4.6 ≤ RDW/ALB ≤ 5.9; and high ratio: RDW/ALB > 5.9.

### Association between RDW/ALB and indexes of The severity of patients’ conditions and kidney dysfunction and All-cause mortality of AKI

We found RDW and ALB were independently associated with 1-month, 3-month, and 12-month all-cause mortality of AKI patients (Supplementary Tables S2 and S3). Table 2 shows the association between RDW/ALB and indexes of the severity of patients’ conditions and kidney dysfunction. Results displayed that RDW/ALB was negatively associated with eGFR, and positively associated with SOFA, SAPSII, and AKI stage (all *p* < 0.001).

**Table 2:** The correlation of indexes of the severity of patients’ conditions and kidney dysfunction with the RDW/ALB ratio.

Variables	RDW/ALB	
	Correlation	*p*
eGFR	−0.109	<0.001
SOFA	0.213	<0.001
SAPSII	0.220	<0.001
AKI stage	0.189	<0.001

RDW/ALB, red blood cell distribution width/albumin; AKI, acute kidney injury; eGFR, estimated glomerular filtration rate; SOFA, Sequential Organ Failure Assessment score; and SAPSII, Simplified Acute Physiology Score II.

Table 3 shows the association between RDW/ALB and all-cause mortality of AKI. In the unadjusted model, moderate and high RDW/ALB ratios were associated with the increased risk of 1-month, 3-month, and 12-month all-cause mortality compared to the low RDW/ALB ratio group (all *p* < 0.001). In model 1, after adjusting age, ethnicity, and gender, similar results were observed (all *p* < 0.001). In model 2, after adjusting more confounders, RDW/ALB was also an independent factor for 1-month, 3-month, and 12-month all-cause mortality in AKI patients, with HR of 1.31 (95%CI: 1.18–1.45), 1.41 (95%CI: 1.30–1.53), and 1.39 (95%CI: 1.30–1.49), respectively (moderate ratio vs. low ratio), and HR of 1.67 (95%CI: 1.49–1.86), 1.77 (95%CI: 1.62–1.93), and 1.76 (95%CI: 1.64–1.90), respectively (high ratio vs. low ratio).

**Table 3:** Hazard ratios (HRs) for all-cause mortality based on RDW/ALB ratios in acute kidney injury (AKI) patients.

RDW/ALB ratio	Unadjusted model		Model 1		Model 2	
	HR (95%CI)	*p*	HR (95%CI)	*p*	HR (95%CI)	*p*
1-month all-cause mortality						
Low	Ref		Ref		Ref	
Moderate	1.58 (1.43–1.75)	<0.001	1.58 (1.43–1.74)	< 0.001	1.31 (1.18–1.45)	<0.001
High	2.34 (2.133–2.57)	<0.001	2.38 (2.16–2.61)	<0.001	1.67 (1.49–1.86)	<0.001
3-month all-cause mortality						
Low	Ref		Ref		Ref	
Moderate	1.72 (1.592–1.86)	<0.001	1.72 (1.60–1.86)	<0.001	1.41 (1.30–1.53)	<0.001
High	2.47 (2.296–2.67)	<0.001	2.55 (2.37–2.74)	<0.001	1.77 (1.62–1.93)	<0.001
12-month all-cause mortality						
Low	Ref		Ref		Ref	
Moderate	1.70 (1.590–1.81)	<0.001	1.71 (1.60–1.82)	<0.001	1.39 (1.30–1.49)	<0.001
High	2.39 (2.243–2.55)	<0.001	2.49 (2.33–2.65)	<0.001	1.76 (1.64–1.90)	<0.001

HR, hazard ratio; RDW/ALB, red blood cell distribution width/albumin; AKI, acute kidney injury; CI, confidence interval.

Low ratio: RDW/ALB < 4.6; moderate ratio: 4.6 ≤ RDW/ALB ≤ 5.9; high ratio: RDW/ALB > 5.9.

Unadjusted model, adjusted for none; Model 1, adjusted for age, ethnicity, and gender; Model 2, adjusted for age, ethnicity, AKI stage, heart rate temperature, SPO2, PCO_2_, Hematocrit percent, lymphocytes, hemoglobin, creatinine, INR, BUN, lactate, Calcium, NLR, Bicarbonate, anion gap, AF, CHF, respiratory failure, diabetes, hypertension, ECI, mechanical ventilation, vasopressors, SOFA, SAPSII, RRT, sepsis, acute pancreatitis, liver cirrhosis, and acute cerebrovascular disease.

### Predictive value of RDW/ALB in the all-cause mortality of AKI patients

**Figure 2:**
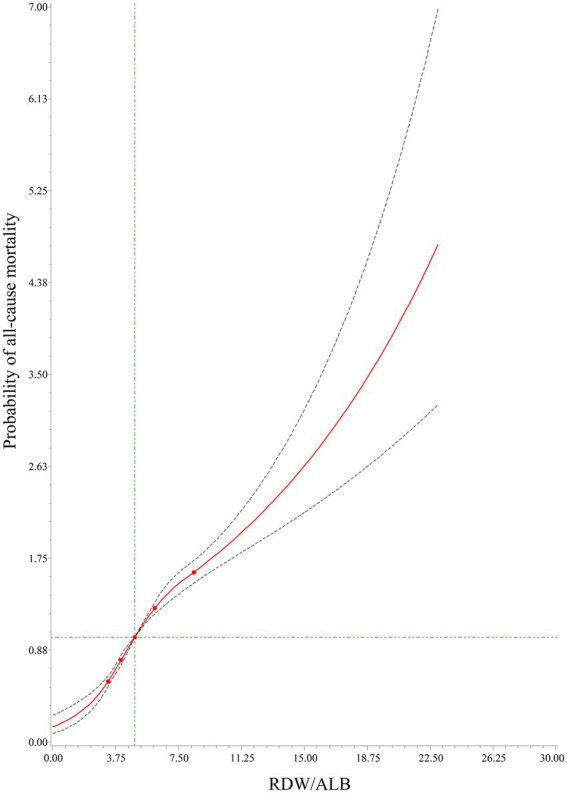
The relationship between the red cell distribution width (RDW)/albumin (ALB) ratio and the crude probability of all-cause mortality using RCS.

**Figure 3:**
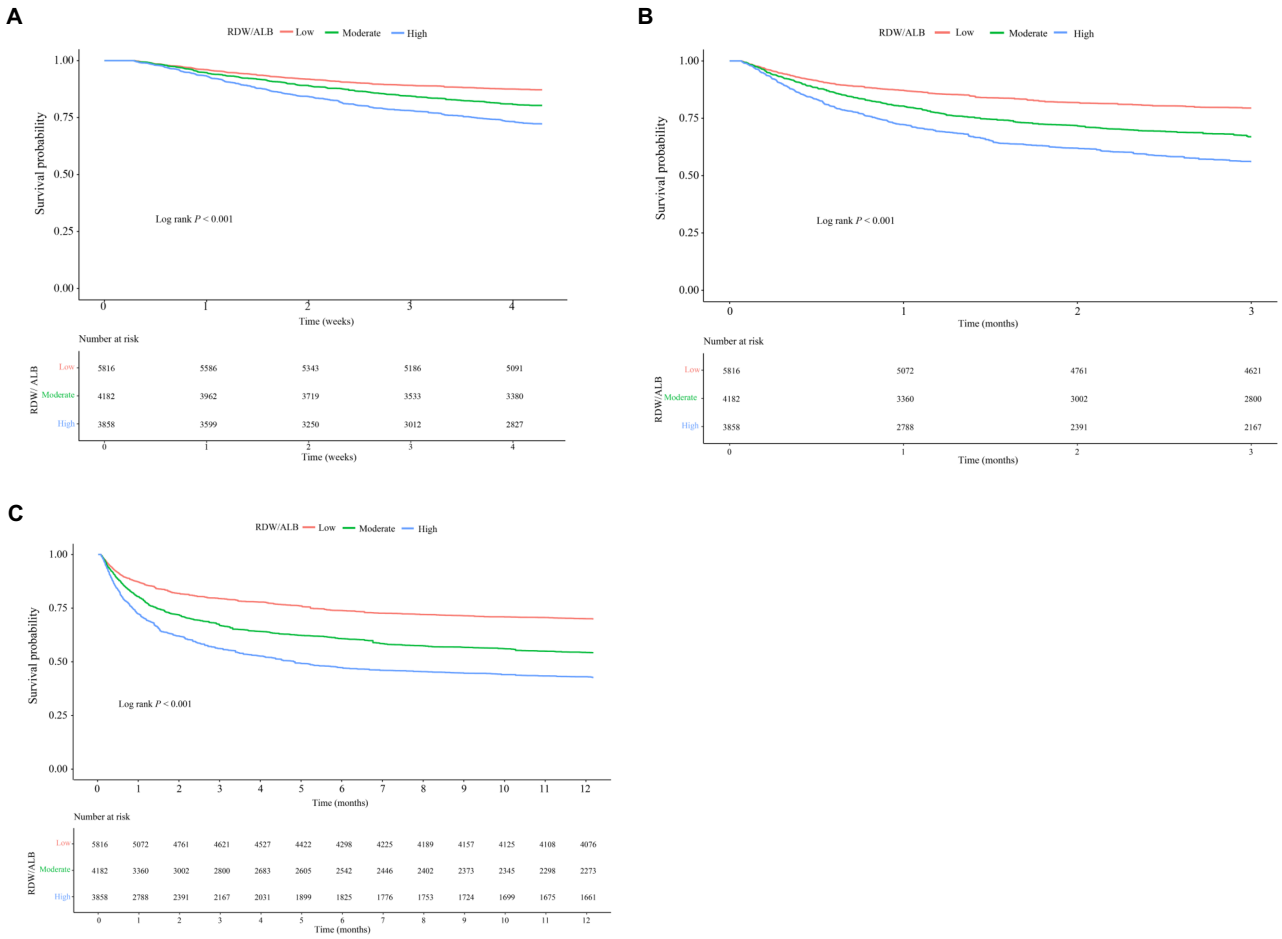
Kaplan–Meier (KM) survival curves of 1-month **(A)**, 3-month **(B)**, and 12-month **(C)** in patients with low, moderate, and high RDW/ALB ratios.

Table 4 demonstrates that the C-index of RDW/ALB for 1-month, 3-month, and 12-month all-cause mortality was 0.728 (95%CI: 0.719–0.737), 0.728 (95%CI: 0.721–0.735), and 0.719 (95%CI: 0.713–0.725), respectively. The DeLong test displayed that the performance of RDW/ALB to predict the risk of 1-month, 3-month, and 12-month all-cause mortality was superior to RDW, ALB, SAPSII, and SOFA (all *p* < 0.05; Table 5). RCS curves showed a linear correlation between the RDW/ALB ratio and the crude probability of all-cause mortality and the probability of all-cause mortality was increased with the increase of the RDW/ALB ratio (Figure 2). Consistently, Figures 3A–C demonstrate that the 1-month, 3-month, and 12-month survival of patients with moderate and high RDW/ALB ratios was lower than those with the low ratio (log rank *p* < 0.001).

**Table 4:** Predictive value of RDW/ALB, RDW, ALB, SAPSII, and SOFA in all-cause mortality of AKI.

Variables	RDW/ALB	RDW	ALB	SAPSII	SOFA
	C-index (95%CI)	C-index (95%CI)	C-index (95%CI)	C-index (95%CI)	C-index (95%CI)
1-month all-cause mortality	0.728 (0.719–0.737)	0.714 (0.704–0.724)	0.710 (0.700–0.720)	0.653 (0.643–0.663)	0.562 (0.551–0.573)
3-month all-cause mortality	0.728 (0.721–0.735)	0.718 (0.710–0.726)	0.711 (0.704–0.719)	0.647 (0.639–0.655)	0.568 (0.559–0.576)
12-month all-cause mortality	0.719 (0.713–0.725)	0.708 (0.701–0.714)	0.701 (0.695–0.708)	0.642 (0.635–0.649)	0.563 (0.555–0.570)

RDW/ALB, red blood cell distribution width/albumin; RDW, red blood cell distribution width; ALB, albumin; SAPSII, Simplified Acute Physiology Score II; SOFA, Sequential Organ Failure Assessment score; C-index, concordance index; and CI, confidence interval.

**Table 5:** Comparison of differences in C-index of RDW/ALB, RDW, ALB, SAPSII, and SOFA.

Variables	RDW/ALB vs. RDW	RDW/ALB vs. ALB	RDW/ALB vs. SAPSII	RDW/ALB vs. SOFA
	*p*	*p*	*p*	*p*
1-month all-cause mortality	0.030	0.005	<0.001	<0.001
3-month all-cause mortality	0.046	0.001	<0.001	<0.001
12-month all-cause mortality	0.011	<0.001	<0.001	<0.001

RDW/ALB, red blood cell distribution width/albumin; RDW, red blood cell distribution width; ALB, albumin; SAPSII, Simplified Acute Physiology Score II; SOFA, Sequential Organ Failure Assessment score.

### Subgroup analysis for the association between RDW/ALB and all-cause mortality based on AKI stage and sepsis

Results of subgroup analysis based on the AKI stage are shown in Table 6. In AKI patients at stage 1, we found moderate and high RDW/ALB ratios were associated with a higher risk of 1-month, 3-month, and 12-month all-cause mortality compared to the low ratio, with *p* < 0.05 for all. The results remained similar in the patients at stage 2. In AKI patients at stage 3, a moderate RDW/ALB ratio increased the risk of 3-month all-cause mortality (HR: 1.30, 95%CI: 1.12–1.52) and 12-month all-cause mortality (HR: 1.34, 95%CI: 1.18–1.53), while there was no significant difference between moderate ratio and 1-month all-cause mortality. A high RDW/ALB ratio showed a positive association with 1-month, 3-month, and 12-month all-cause mortality, with HR of 1.46 (95%CI: 1.21–1.76), 1.59 (95%CI: 1.36–1.85), 1.66 (95%CI: 1.46–1.90), respectively.

**Table 6:** Hazard ratios (HRs) for the all-cause mortality of AKI patients based on AKI stage.

RDW/ALB ratio	1-month all-cause mortality		3-month all-cause mortality		12-month all-cause mortality	
	HR (95%CI)	*p*	HR (95%CI)	*p*	HR (95%CI)	*p*
AKI stage						
Stage 1						
Low	Ref		Ref		Ref	
Moderate	1.40 (1.09–1.79)	0.008	1.41 (1.17–1.70)	<0.001	1.40 (1.20–1.62)	<0.001
High	2.21 (1.68–2.90)	<0.001	2.27 (1.85–2.80)	<0.001	2.26 (1.90–2.68)	<0.001
Stage 2						
Low	Ref		Ref		Ref	
Moderate	1.41 (1.21–1.64)	<0.001	1.51 (1.34–1.69)	<0.001	1.43 (1.30–1.58)	<0.001
High	1.77 (1.51–2.08)	<0.001	1.80 (1.59–2.04)	<0.001	1.71 (1.54–1.91)	<0.001
Stage 3						
Low	Ref		Ref		Ref	
Moderate	1.12 (0.93–1.35)	0.251	1.30 (1.12–1.52)	<0.001	1.34 (1.18–1.53)	<0.001
High	1.46 (1.21–1.76)	<0.001	1.59 (1.36–1.85)	<0.001	1.66 (1.46–1.90)	<0.001

HR, hazard ratio; AKI, acute kidney injury; RDW/ALB, red blood cell distribution width/albumin; and CI, confidence interval.

Low ratio: RDW/ALB < 4.6; moderate ratio: 4.6 ≤ RDW/ALB ≤ 5.9; high ratio: RDW/ALB > 5.9.

Results of subgroup analysis based on sepsis are shown in Supplementary Table S4. In patients with sepsis, we found that moderate and high RDW/ALB ratios were associated with a higher risk of 1-month, 3-month, and 12-month all-cause mortality than the low RDW/ALB ratio (all *p* < 0.001). Similar results were found in patients without sepsis (all *p* < 0.001).

### Association and predictive value of RDW/ALB for the risk of RRT In AKI patients

Supplementary Table S5 shows the significant association between moderate and high RDW/ALB ratios and the risk of RRT in the unadjusted model. After adjusting age, ethnicity, and gender, the results remained similar. Further adjusting for more confounders, a high RDW/ALB ratio was found to be associated with the risk of RRT (HR: 1.26, 95%CI: 1.08–1.47). The RCS curve displayed a linear relationship between the RDW/ALB ratio and the crude probability of RRT (Supplementary Figure S2). Moreover, we found the good performance of RDW/ALB in predicting the risk of RRT in AKI patients (C-index: 0.883, 95%CI: 0.876–0.890; Supplementary Table S6).

## Discussion

In our study, we found that an increase in RDW/ALB was associated with a higher risk of all-cause mortality in critically ill patients with AKI. This association was also observed in AKI patients at stage 1, stage 2, and stage 3, and in patients with or without sepsis. The predictive performance of RDW/ALB in the risk of all-cause mortality was superior to RDW, ALB, SAPSII, and SOFA. Moreover, we found RDW/ALB was associated with RRT and performed well in predicting the risk of RRT.

Acute kidney injury (AKI) is defined as a rapid increase in SCr, a decrease in urine output, or both (23). More than half of ICU patients experience AKI, which brings health and medical burdens (1). Inflammation is initiated by the interaction of the host and pathogen and is a main pathogenesis to develop fatal consequences in patients, including in AKI patients (30, 31). Based on inflammatory response as a main mechanism, identifying new inflammatory indicators or previously known indicators could be valuable to predict clinical outcomes.

Red cell distribution width (RDW) is increased in response to inflammatory stimuli (9), and increased RDW is reported to be associated with unfavorable outcomes in many inflammatory diseases, such as pneumonia (32), sepsis (33), and ARDS (34). Previous studies have reported a positive association between RDW and all-cause mortality of many critically ill conditions (35–37). The elaborated mechanisms of RDW increase in critically ill patients remain to be determined; inflammation is induced to generate premature erythrocytes of premature erythrocytes from the bone marrow (38). Moreover, oxidative stress overproduces reactive oxygen species, which also stimulates erythropoiesis, resulting in anisocytosis and the increase of RDW (39). ALB is also an indicator related to inflammation (40). In inflammatory states, microvascular permeability and ALB escape is increased; this expands interstitial space and increases the distribution volume of ALB (40). In addition, the half-life of ALB is shown to shorten, reducing the total mass of ALB (40). These two factors result in hypoalbuminemia in many critically ill patients, and over 50% of them have ALB concentrations lower than normal values (40, 41). Importantly, a low ALB concentration is associated with higher mortality (41). Existing evidence has supported the accuracy of combined inflammation-related indexes in the prediction of mortality (14, 20). Previously, studies have reported a positive relationship between RDW/ALB and all-cause mortality of critically ill patients (19–21). Our findings were consistent with previous studies that RDW/ALB was associated with all-cause mortality of ICU patients with AKI, with a higher ratio indicating a higher risk of mortality. Existing studies showed that AKI stages were independently associated with the mortality of ICU patients (42, 43). In our study, we found moderate and high RDW/ALB ratios increased the risk of all-cause mortality in each AKI stage. To make our results more reliable, we also performed subgroups based on sepsis since inflammation may play a role in determining RDW and albumin status. Results showed that a higher RDW/ALB ratio was associated with a higher risk of all-cause mortality in patients with or without sepsis.

Our study also found a better predictive performance of RDW/ALB in the risk of all-cause mortality than RDW, ALB, SAPSII, and SOFA. RDW and ALB have been previously reported to independently predict the prognosis of patients with serious diseases (44, 45). Our study displayed the association between RDW or ALB and the risk of all-cause mortality in AKI patients, while the performance of RDW/ALB was better than RDW and ALB in the risk of all-cause mortality. The SOFA score aims to quantitatively assess organ dysfunction over time, and the association between the quantification of the SOFA score and mortality is inevitable (46). Many studies have reported the predictive value of the SOFA score for the risk of mortality in critically ill patients (47–49). SAPSII scoring system contains age, physiological variables, and several complications, which can estimate the death risk of critically ill patients (50, 51). SOFA and SAPSII have been recommended to evaluate the prognosis outcome of ICU patients who experience AKI; however, SOFA presents lower accuracy and SAPSII requires complex calculations (52, 53). RDW/ALB was easily calculated in clinical practice and showed better performance than SAPSII and SOFA. Our study indicated that RDW/ALB may be a simple and effective predictor for the risk of all-cause mortality in critically ill patients with AKI.

Acute kidney injury requiring RRT is a serious clinical disorder in the ICU, and the use of RRT is increasing, with 5–10% of ICU patients requiring RRT (54, 55). In this study, we also explored the association and predictive performance of the RDW/ALB ratio in the risk of RRT. Results showed that a high RDW/ALB ratio was associated with a higher risk of RRT. Moreover, we found RDW/ALB performed well to predict the risk of RRT, indicating that RDW/ALB may also be an effective predictor for the risk of RRT in critically ill patients with AKI.

Our study used a large sample size to explore the association between RDW/ALB and the risk of all-cause mortality and RRT and to investigate the ability of RDW/ALB to predict the risk of all-cause mortality and RRT in critically ill patients with AKI. However, there are some limitations in this study. First, this is a retrospective study, the selective bias is inevitable, making it difficult to generalize the findings to all AKI patients. Future studies are needed to externally validate our findings. Second, our data were extracted from the MIMIC-III database. Due to the defects of the database, treatment and care conditions out of hospital (potential confounders) were difficult to obtain. Third, the RDW/ALB ratio was calculated at baseline, and whether monitoring the RDW/ALB ratio might better predict all-cause mortality of AKI patients’ needs to be assessed. Fourth, the causes of AKI may be different. We have provided some of the causes of AKI and performed subgroup analysis based on sepsis. Due to the other specific causes for AKI not being recorded in the MIMIC-III database, we could not carry out further analyses.

## Conclusion

In conclusion, our study showed that RDW/ALB was associated with the risk of all-cause mortality and RRT, and performed well to predict the risk of all-cause mortality and RRT in AKI patients admitted to the ICU. Our findings indicated that clinicians may use RDW/ALB to predict the risk of all-cause mortality and RRT of ICU patients with AKI, which may help them take adequate interventions to improve the prognosis of high-risk patients.

## Data availability statement

Publicly available datasets were analyzed in this study. This data can be found at: MIMIC-III database, https://mimic.physionet.org/iii/.

## Ethics statement

The studies involving human participants were reviewed and approved by Beth Israel Deaconess Medical Center (Boston, MA, United States) and the Massachusetts Institute of Technology (Cambridge, MA, United States). The patients/participants provided their written informed consent to participate in this study.

## Author contributions

CG and LP designed the study. CG wrote the manuscript, collected, analyzed, and interpreted the data. LP critically reviewed and edited the manuscript. All authors contributed to the article and approved the submitted version.

## Funding

This work is supported by Hunan Clinical Medical Technology Innovation Guidance Project 2021SK53524.

## Conflict of interest

The authors declare that the research was conducted in the absence of any commercial or financial relationships that could be construed as a potential conflict of interest.

## Publisher’s note

All claims expressed in this article are solely those of the authors and do not necessarily represent those of their affiliated organizations, or those of the publisher, the editors and the reviewers. Any product that may be evaluated in this article, or claim that may be made by its manufacturer, is not guaranteed or endorsed by the publisher.
