# Supplementary figures and images for: Association and prediction of red blood cell distribution width to albumin ratio in all-cause mortality of acute kidney injury in critically ill patients
Source: Front Med (Lausanne). 2023 Mar 9;10:1047933. doi: 10.3389/fmed.2023.1047933 (PMC10034203; doi:10.3389/fmed.2023.1047933)

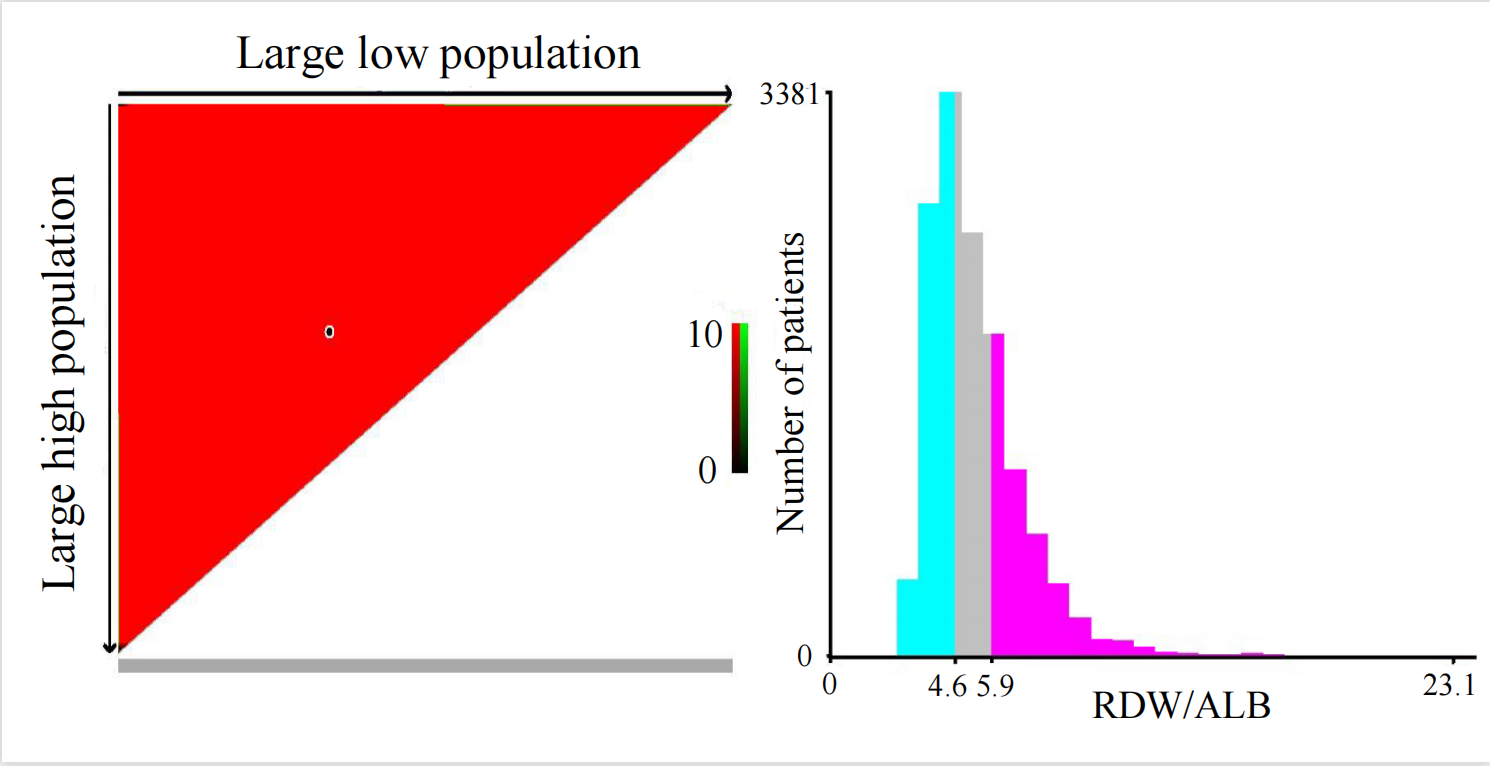

Supplement: Supplementary file 1 [file Image_1.TIF]

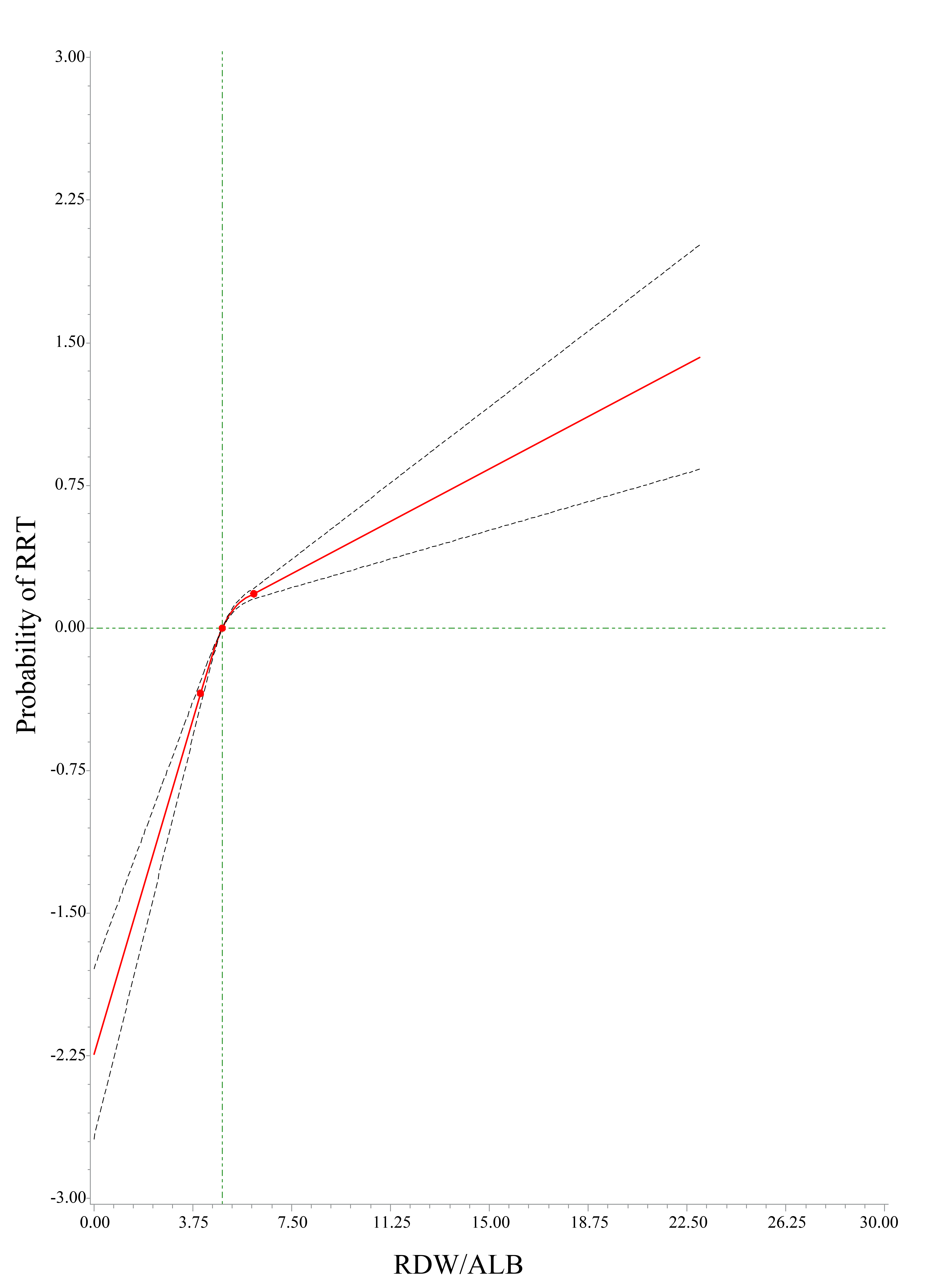

Supplement: Supplementary file 2 [file Image_2.TIF]
