# Supplementary material for: Association and prediction of red blood cell distribution width to albumin ratio in all-cause mortality of acute kidney injury in critically ill patients
Source: Front Med (Lausanne). 2023 Mar 9;10:1047933. doi: 10.3389/fmed.2023.1047933 (PMC10034203; doi:10.3389/fmed.2023.1047933)
Supplement: Supplementary file 3 [file Table_1.docx]

**Supplementary table S1 Sensitivity analysis for multiple imputation of missing data**

| **Variables** | **Missing, n (%)** | **Before imputation** | **After imputation** | **statistics** | ***P*** |
| --- | --- | --- | --- | --- | --- |
| Lactate, M (Q_1_, Q_3_) | 1313 (9.48) | 1.80 (1.30, 2.90) | 1.80 (1.30, 2.90) | Z = 0.943 | 0.346 |
| ALT, M (Q_1_, Q_3_) | 916 (6.61) | 27.00 (16.00, 54.00) | 27.00 (16.00, 54.00) | Z = 0.339 | 0.735 |
| AST, M (Q_1_, Q_3_) | 923 (6.66) | 37.00 (22.00, 78.00) | 37.00 (22.00, 77.00) | Z = 0.699 | 0.484 |
| PCO_2_, M (Q_1_, Q_3_) | 870 (6.28) | 41.00 (36.00, 49.00) | 41.00 (35.00, 49.00) | Z = 1.299 | 0.194 |
| Temperature, Mean ± SD | 71 (0.51) | 36.61 ± 1.11 | 36.61 ± 1.11 | t = -0.02 | 0.984 |
| INR, M (Q_1_, Q_3_) | 48 (0.35) | 1.30 (1.10, 1.60) | 1.30 (1.10, 1.60) | Z = 0.114 | 0.909 |
| Calcium, Mean ± SD | 15 (0.11) | 8.44 ± 1.02 | 8.44 ± 1.02 | t = -0.01 | 0.993 |
| SPO_2_, Mean ± SD | 9 (0.06) | 96.80 ± 5.50 | 96.80 ± 5.50 | t = 0.00 | 0.997 |
| Heart rate, Mean ± SD | 8 (0.06) | 92.75 ± 21.01 | 92.75 ± 21.01 | t = 0.01 | 0.995 |
| SBP, Mean ± SD | 8 (0.06) | 123.36 ± 27.09 | 123.35 ± 27.08 | t = 0.02 | 0.981 |
| DBP, M (Q_1_, Q_3_) | 8 (0.06) | 62.00 (52.00, 74.00) | 62.00 (52.00, 74.00) | Z = 0.018 | 0.985 |
| Respiratory rate, M (Q_1_, Q_3_) | 8 (0.06) | 19.00 (14.00, 24.00) | 19.00 (14.00, 24.00) | Z = -0.028 | 0.978 |
| ECI, M (Q_1_, Q_3_) | 5 (0.04) | 12.00 (5.00, 20.00) | 12.00 (5.00, 20.00) | Z = 0.034 | 0.973 |

Abbreviation: ALT, alanine aminotransferase; AST, aspartate aminotransferase; PCO_2_, partial pressure of carbon dioxide; INR, international normalized ratio; SPO_2_, saturation of peripheral oxygen; SBP, systolic blood pressure; DBP, diastolic blood pressure; ECI, Elixhauser Comorbidity Index.

**Supplementary table S2 HRs for all-cause mortality of AKI based on RDW groups**

| **Variables** | **Unadjusted model** | | **Model 1** | | **Model 2** | |
| --- | --- | --- | --- | --- | --- | --- |
|  | **HR (95%CI)** | ***P*** | **HR (95%CI)** | ***P*** | **HR (95%CI)** | ***P*** |
| **1-month all-cause mortality** |  |  |  |  |  |  |
| Low RDW level | Ref |  | Ref |  | Ref |  |
| Moderate RDW level | 1.49 (1.34-1.66) | < 0.001 | 1.43 (1.29-1.58) | < 0.001 | 1.30 (1.16-1.45) | < 0.001 |
| High RDW level | 2.28 (2.08-2.49) | < 0.001 | 2.30 (2.11-2.51) | < 0.001 | 1.73 (1.56-1.92) | < 0.001 |
| **3-month all-cause mortality** |  |  |  |  |  |  |
| Low RDW level | Ref |  | Ref |  | Ref |  |
| Moderate RDW level | 1.48 (1.37-1.61) | < 0.001 | 1.42 (1.31-1.54) | < 0.001 | 1.29 (1.18-1.40) | < 0.001 |
| High RDW level | 2.39 (2.23-2.56) | < 0.001 | 2.42 (2.26-2.60) | < 0.001 | 1.80 (1.66-1.95) | < 0.001 |
| **12-month all-cause mortality** |  |  |  |  |  |  |
| Low RDW level | Ref |  | Ref |  | Ref |  |
| Moderate RDW level | 1.47 (1.37-1.57) | < 0.001 | 1.40 (1.30-1.50) | < 0.001 | 1.25 (1.17-1.34) | < 0.001 |
| High RDW level | 2.38 (2.24-2.52) | < 0.001 | 2.40 (2.27-2.55) | < 0.001 | 1.72 (1.60-1.84) | < 0.001 |

Abbreviation: HR, hazard ratio; AKI, acute kidney injury; RDW, red blood cell distribution width; CI, confidence interval.

Note: low RDW level (RDW < 14.4), moderate RDW level (14.4 ≤ RDW ≤ 15.7), high RDW level (RDW > 15.7).

Unadjusted model, adjusted for none;

Model 1, adjusted for age, ethnicity, and gender;

Model 2 adjusted for age, ethnicity, AKI stage, heart rate, temperature, SPO_2_, PCO_2_, hematocrit percent, lymphocytes, hemoglobin, creatinine, INR, BUN, lactate, calcium, NLR, bicarbonate, anion gap, AF, CHF, respiratory failure, diabetes, hypertension, ECI, mechanical ventilation, vasopressors, SOFA, SAPSII, RRT, sepsis, acute pancreatitis, liver cirrhosis, and acute cerebrovascular disease.

**Supplementary table S3 HRs for all-cause mortality of AKI based on ALB groups**

| **Variables** | **Unadjusted model** | | **Model 1** | | **Model 2** | |
| --- | --- | --- | --- | --- | --- | --- |
|  | **HR (95%CI)** | ***P*** | **HR (95%CI)** | ***P*** | **HR (95%CI)** | ***P*** |
| **1-month all-cause mortality** |  |  |  |  |  |  |
| Low ALB level | Ref |  | Ref |  | Ref |  |
| Moderate ALB level | 0.83 (0.76-0.90) | < 0.001 | 0.82 (0.75-0.90) | < 0.001 | 0.89 (0.81-0.97) | 0.011 |
| High ALB level | 0.53 (0.48-0.59) | < 0.001 | 0.52 (0.48-0.58) | < 0.001 | 0.70 (0.63-0.78) | < 0.001 |
| **3-month all-cause mortality** |  |  |  |  |  |  |
| Low ALB level | Ref |  | Ref |  | Ref |  |
| Moderate ALB level | 0.81 (0.75-0.86) | < 0.001 | 0.80 (0.74-0.85) | < 0.001 | 0.87 (0.81-0.94) | < 0.001 |
| High ALB level | 0.54 (0.50-0.58) | < 0.001 | 0.53 (0.49-0.57) | < 0.001 | 0.71 (0.65-0.77) | < 0.001 |
| **12-month all-cause mortality** |  |  |  |  |  |  |
| Low ALB level | Ref |  | Ref |  | Ref |  |
| Moderate ALB level | 0.85 (0.80-0.90) | < 0.001 | 0.83 (0.78-0.88) | < 0.001 | 0.89 (0.84-0.95) | < 0.001 |
| High ALB level | 0.58 (0.55-0.62) | < 0.001 | 0.56 (0.53-0.60) | < 0.001 | 0.73 (0.67-0.78) | < 0.001 |

Abbreviation: HR, hazard ratio; AKI, acute kidney injury; ALB, albumin; CI, confidence interval.

Note: low ALB level (ALB < 2.6), moderate ALB level (2.6 ≤ ALB ≤ 3.3), high ALB level (ALB > 3.3).

Unadjusted model, adjusted for none;

Model 1, adjusted for age, ethnicity, and gender;

Model 2 adjusted for age, ethnicity, AKI stage, heart rate, temperature, SPO_2_, PCO_2_, hematocrit percent, lymphocytes, hemoglobin, creatinine, INR, BUN, lactate, calcium, NLR, bicarbonate, anion gap, AF, CHF, respiratory failure, diabetes, hypertension, ECI, mechanical ventilation, vasopressors, SOFA, SAPSII, RRT, sepsis, acute pancreatitis, liver cirrhosis, and acute cerebrovascular disease.

**Supplementary table S4** **HRs for the all-cause mortality of AKI patients with or without sepsis based on RDW/ALB ratio**

| **RDW/ALB ratio** | **No sepsis** | | **Sepsis** | |
| --- | --- | --- | --- | --- |
|  | **HR (95%CI)** | ***P*** | **HR (95%CI)** | ***P*** |
| **1-month all-cause mortality** |  |  |  |  |
| Low | Ref |  | Ref |  |
| Moderate | 1.26 (1.11-1.43) | < 0.001 | 1.54 (1.27-1.87) | < 0.001 |
| High | 1.59 (1.39-1.81) | < 0.001 | 2.14 (1.75-2.62) | < 0.001 |
| **3-month all-cause mortality** |  |  |  |  |
| Low | Ref |  | Ref |  |
| Moderate | 1.39 (1.26-1.53) | < 0.001 | 1.61 (1.38-1.89) | < 0.001 |
| High | 1.73 (1.56-1.91) | < 0.001 | 2.03 (1.71-2.40) | < 0.001 |
| **12-month all-cause mortality** |  |  |  |  |
| Low | Ref |  | Ref |  |
| Moderate | 1.40 (1.29-1.52) | < 0.001 | 1.50 (1.31-1.72) | < 0.001 |
| High | 1.82 (1.67-1.99) | < 0.001 | 1.78 (1.53-2.08) | < 0.001 |

Abbreviation: HR, hazard ratio; AKI, acute kidney injury; RDW/ALB, red blood cell distribution width/albumin; CI, confidence interval.

**Supplementary table S5 HRs for the risk of RRT in AKI patients based on RDW/ALB ratio**

| **RDW/ALB ratio** | **Unadjusted model** | | **Model 1** | | **Model 2** | |
| --- | --- | --- | --- | --- | --- | --- |
|  | **HR (95%CI)** | ***P*** | **HR (95%CI)** | ***P*** | **HR (95%CI)** | ***P*** |
| Risk of RRT |  |  |  |  |  |  |
| Low | Ref |  | Ref |  | Ref |  |
| Moderate | 1.74 (1.56-1.95) | < 0.001 | 1.77 (1.58-1.98) | < 0.001 | 1.11 (0.96-1.29) | 0.168 |
| High | 2.19 (1.96-2.45) | < 0.001 | 2.23 (2.00-2.50) | < 0.001 | 1.26 (1.08-1.47) | 0.003 |

Abbreviation: HR, hazard ratio; RRT, renal replacement therapy; AKI, acute kidney injury; RDW/ALB, red blood cell distribution width/albumin; CI, confidence interval.

Note: low ratio: RDW/ALB < 4.6; moderate ratio: 4.6 ≤ RDW/ALB ≤ 5.9; high ratio: RDW/ALB > 5.9.

Unadjusted model, adjusted for none;

Model 1, adjusted for age, ethnicity, and gender;

Model 2 adjusted for age, AKI stage, PCO_2_, lymphocytes, platelet, hematocrit, creatinine, BUN, bicarbonate, calcium, ALT, AST, anion gap, eGFR, NLR, AF, CHF, ECI, SOFA, mechanical ventilation, vasopressors, sepsis, acute pancreatitis, liver cirrhosis, acute cerebrovascular disease, and cardiogenic shock.

**Supplementary table S6 Predictive value of RDW/ALB, RDW, ALB, SAPSII, and SOFA in the risk of RRT of AKI patients**

| **Variables** | **RDW/ALB** | **RDW** | **ALB** | **SAPSII** | **SOFA** |
| --- | --- | --- | --- | --- | --- |
|  | **C-index (95%CI)** | **C-index (95%CI)** | **C-index (95%CI)** | **C-index (95%CI)** | **C-index (95%CI)** |
| Risk of RRT | 0.883 (0.876-0.890) | 0.878 (0.871-0.886) | 0.879 (0.871-0.886) | 0.679 (0.667-0.691) | 0.730 (0.719-0.742) |

Abbreviation: RDW/ALB, red blood cell distribution width/albumin; RDW, red blood cell distribution width; ALB, albumin; SAPSII, Simplified Acute Physiology Score II; SOFA, Sequential Organ Failure Assessment score; RRT, renal replacement therapy; AKI, acute kidney injury; C-index, concordance index; CI, confidence interval.
